# Enhancement of the electrochemical performance of the spinel structure LiNi_0.5-x_Ga_x_Mn_1.5_O_4_ cathode material by Ga doping

**DOI:** 10.1186/s11671-018-2666-3

**Published:** 2018-08-22

**Authors:** Lifang Lan, Sheng Li, Jun Li, Lu Lu, Yan Lu, Si Huang, Shuaijun Xu, Chunyang Pan, Fenghua Zhao

**Affiliations:** Faculty of Chemical Engineering and Light Industry, Guangzhou Higher Education Mega Center, Guangdong University of Technology, No. 100 Waihuan xi Road, Panyu District, Guangzhou, 510006 Guangdong China

**Keywords:** Ga doping, LiNi_0.5-x_Ga_x_Mn_1.5_O_4_, Lithium-ion batteries, Electrochemical performance, Passivation layer

## Abstract

A sol-gel method was adopted to obtain LiNi_0.5-x_Ga_x_Mn_1.5_O_4_ (*x* = 0, 0.04, 0.06, 0.08, 0.1) samples. The effect of Ga doping on LiNi_0.5_Mn_1.5_O_4_ and its optimum content were investigated, and the electrochemical properties at room temperature and at a high temperature were discussed. The structural, morphological, and vibrational features of LiNi_0.5-x_Ga_x_Mn_1.5_O_4_ (*x* = 0, 0.04, 0.06, 0.08, 0.1) compounds were characterized by X-ray diffraction (XRD), scanning electron microscopy (SEM), and Fourier transform infrared spectroscopy (FT-IR). The XRD results demonstrate that all samples have a disordered spinel structure with a space group of Fd3m, and Ga doping restrains the formation of the Li_x_Ni_1-x_O secondary phase. FT-IR analysis reveals that Ga doping increases the degree of cation disorder. The SEM results reveal that all samples possess a fine spinel octahedron crystal. The electrochemical performance of the samples was investigated by galvanostatic charge/discharge tests, dQ/dV plots, and electrochemical impedance spectroscopy (EIS). The LiNi_0.44_Ga_0.06_Mn_1.5_O_4_ sample with the optimum content shows a superior rate performance and cycle stability after Ga doping, especially at a high discharge rate and high temperature. In addition, the LiNi_0.44_Ga_0.06_Mn_1.5_O_4_ sample retained 98.3% of its initial capacity of 115.7 mAhg^−1^ at the 3 C discharge rate after 100 cycles, whereas the pristine sample delivered a discharge capacity of 87.3 mAhg^−1^ at 3 C with a capacity retention of 80% at the 100th cycle. Compared with the pristine material, the LiNi_0.44_Ga_0.06_Mn_1.5_O_4_ sample showed a high capacity retention from 74 to 98.4% after 50 cycles at a 1 C discharge rate and 55 °C.

## Background

With the increasing application of lithium-ion batteries, their requirements are also increasing. Batteries with a long cycle life, high energy density, and low cost could meet the needs of consumers. Spinel LiNi_0.5_Mn_1.5_O_4_ (LNMO) has captured the attention of researchers in related fields [1] due to its high working potential [2], low cost [3], and high energy density [4]of 658 Wh kg^−1^. All of the advantages of LiNi_0.5_Mn_1.5_O_4_ are due to its three-dimensional lithium-ion diffusion path and high working voltage [5].

However, spinel LiNi_0.5_Mn_1.5_O_4_ materials also have several issues to be solved. Firstly, a Li_x_Ni_1-x_O secondary phase forms during the preparation process of spinel LiNi_0.5_Mn_1.5_O_4_ materials [6]. Secondly, the electrolyte is prone to decomposition at high working voltage (4.7 V) (vs Li/Li^+^) [1], which triggers a decrease in capacity and poor electrochemical performance.

Numerous attempts have been proposed to improve the electrochemical performance. Elemental doping and the application of coatings, such as Cr [7], Mg [8], Y [9], Ce [10], Al [11], Cu [12], and Ga [13] doping, as well as BiFeO_3_ [14] and Al_2_O_3_ [15] coatings, could enhance the cycle life or rate performance of LiNi_0.5_Mn_1.5_O_4_ samples to different degrees. For instance, Ce-doped LiNi_0.5_Mn_1.5_O_4_ can improve the cycling stability (94.51% capacity retention after 100 cycles) [10], Al_2_O_3_ coating layer reduces side reactions occurred. The first investigation of the substitution of Mn sites by Ga in the LiMn_2_O_4_ spinel structure was reported by Liu et al. They found that Ga doping can inhibit the Jahn-Teller cooperative distortion of the spinel structure [16]. In 2011, Shin et al. published a paper in which they determined that Ga-doped samples can form a more stable interface and stabilize the spinel structure due to the presence of Ga on the surface of the samples [13]. One year later, Shin [17] synthesized LiMn_1.5_Ni_0.5 − x_M_x_O_4_ (M = Cr, Fe, and Ga) by a hydroxide precursor method and found that the Ga-doped sample and the pristine sample exhibit a decline in the rate capability after annealing at 700 °C. Moreover, they also found that the poor rate capability was caused by the extensive segregation of Ga^3+^ after annealing. Wei Wu et al. published a paper in which they put forward that the characteristic of the solid-state method is that the particles were nonuniform in size and distribution [9]. Sol-gel method is in favor of the formation of well-crystallized octahedrons and a narrow particle distribution according to Wang [18]. However, little attention has been devoted to systematically investigating the rate capacity and electrical conductivity at different Ga-doping contents and the role of Ga at high temperatures. To understand how the Ga-doping concentrations influence the electrochemical properties in detail and to investigate suitable Ga-doped contents of LiNi_0.5_Mn_1.5_O_4_ materials, samples with various Ga-doping concentrations were prepared by a sol-gel method for the first time. The structure, morphology, and electrochemical performance of the samples were investigated systematically.

## Results and discussion

### Structural and morphological analysis

**Fig. 1:**
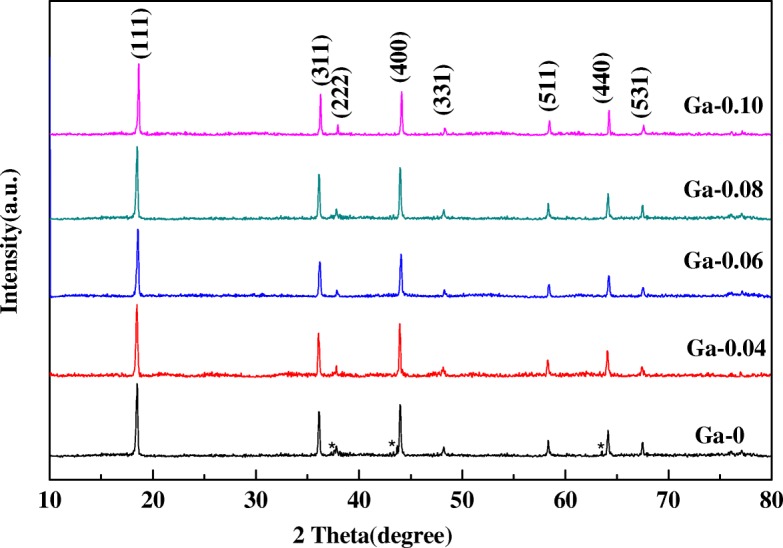
XRD patterns of the LiNi_0.5-x_Ga_x_Mn_1.5_O_4_ (*x* = 0, 0.04, 0.06, 0.08, 0.1) samples

XRD patterns of the LiNi_0.5-x_Ga_x_Mn_1.5_O_4_ (*x* = 0, 0.04, 0.06, 0.08, 0.1) specimens are provided in Fig. 1, which clearly shows that the major diffraction peaks of the samples are consistent with the cards (JCPDS No. 80-2162) for the disordered spinel structure with space group Fd3m. Another paramount finding was that additional diffraction peaks appeared at 37.4°, 43.7°, and 63.8° (marked with an *) in the LiNi_0.5_Mn_1.5_O_4_ sample in addition to the major diffraction peaks, which should be assigned to the Li_x_Ni_1-x_O secondary phase. The finding agrees with results reported previously, in which the formation of the Li_x_Ni_1-x_O secondary phase should be ascribed to high-temperature sintering, and it was considered to decrease the amount of active material [19]. The existence of the Li_x_Ni_1-x_O secondary phase could inhibit the Li^+^ ion diffusion according to Wu [9]. However, no additional secondary phase was detected in the Ga-doped samples, suggesting that Ga doping could inhibit the formation of Li_x_Ni_1-x_O impure phases and provide a single phase.

According to a report that the intensity ratio of I_311_/I_400_ peaks could reflect the stability of the structure [20], whereby a positive correlation exists between the value of I_311_/I_400_ and the stability of the structure. The intensity ratios of the I_311_/I_400_ peaks for the LiNi_0.5-x_Ga_x_Mn_1.5_O_4_ (*x* = 0, 0.04, 0.06, 0.08, 0.1) samples are 0.8636, 0.9115, 0.9216, 0.9097, and 0.8966 (as listed Table 1), respectively. According to the value of I_311_/I_400_, we can infer that Ga doping can promote structural stability. In addition, Table 1 clearly shows the rise of the intensity ratio of I_311_/I_400_ peaks and then a decline as the Ga-doping content further increased; the ratio reached a maximum in the LiNi_0.44_Ga_0.06_Mn_1.5_O_4_ sample, suggesting that this sample has the most stable structure. The finding is consistent with the cyclic performance curve at a high rate and high temperature.

**Table 1:** I_311_/I_400_ values of the LiNi_0.5-x_Ga_x_Mn_1.5_O_4_ (*x* = 0, 0.04, 0.06, 0.08, 0.1) samples

Samples (LiNi_0.5-x_Ga_x_Mn_1.5_O_4_)	I_311_/I_400_
Ga-0	0.8636
Ga-0.04	0.9115
Ga-0.06	0.9216
Ga-0.08	0.9097
Ga-0.1	0.8966

**Fig. 2:**
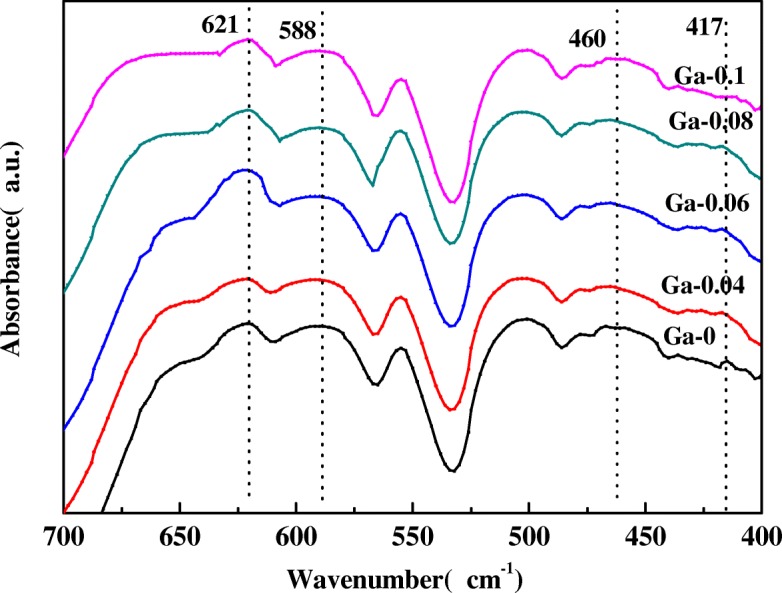
FT-IR spectra of the LiNi_0.5-x_Ga_x_Mn_1.5_O_4_ (*x* = 0, 0.04, 0.06, 0.08, 0.1) samples

To further investigate the space group of the LiNi_0.5-x_Ga_x_Mn_1.5_O_4_ (*x* = 0, 0.04, 0.06, 0.08, 0.1) samples, FT-IR spectroscopy (shown in Fig. 2) was performed in the range of 400–700 cm^−1^. The key to determining the disordered Fd3m space group and the ordered P4_3_32 space group is the disordering degree of the Ni^2+^ and Mn^4+^ in the spinel structure. The bands at 588 and 621 cm^−1^ correspond to the Ni-O bond and the Mn-O bond, respectively. A stronger peak intensity at 621 cm^−1^ rather than at 588 cm^−1^ is characteristic of the Fd3m structure [21]. Kunduraci et al. [22] published a paper in which they observed that the lower the value of I_588_/I_621_ was, the higher the disordering degree of the Mn^4+^ and Ni^2+^ ions in the spinel structure would be. The high degree of cation disorder leads to high conductivity. We calculated the intensity ratios of I_588_/I_621_ as 0.9524, 0.9187, 0.708, 0.8525, and 0.9263 (as listed in Table 2) for Ga-0, Ga-0.04, Ga-0.06, Ga-0.08, and Ga-0.1 samples, respectively. Interestingly, the value of I_588_/I_621_ first decreases and then increases with increasing Ga content, indicating the rise in the degree of cation disorder and then a decline following the increase of Ga-doping content. Ga-0.06 shows the lowest value of I_588_/I_621_, suggesting that it has the highest degree of cation disorder. The value of I_588_/I_621_ is less than 1, characteristic of the disordered Fd3m structure [21], which is consistent with the result of the above XRD analysis. Compared to the ordered P_4_332 structure, the disordered Fd3m structure showed better electrochemical properties than those of the ordered P_4_332 structure [23].

**Table 2:** I_588_/I_621_ values of LiNi_0.5-x_Ga_x_Mn_1.5_O_4_ (*x* = 0, 0.04, 0.06, 0.08, 0.1) samples

Samples (LiNi_0.5-x_Ga_x_Mn_1.5_O_4_)	I_588_/I_621_
Ga-0	0.9524
Ga-0.04	0.9187
Ga-0.06	0.7080
Ga-0.08	0.8525
Ga-0.1	0.9263

**Fig. 3:**
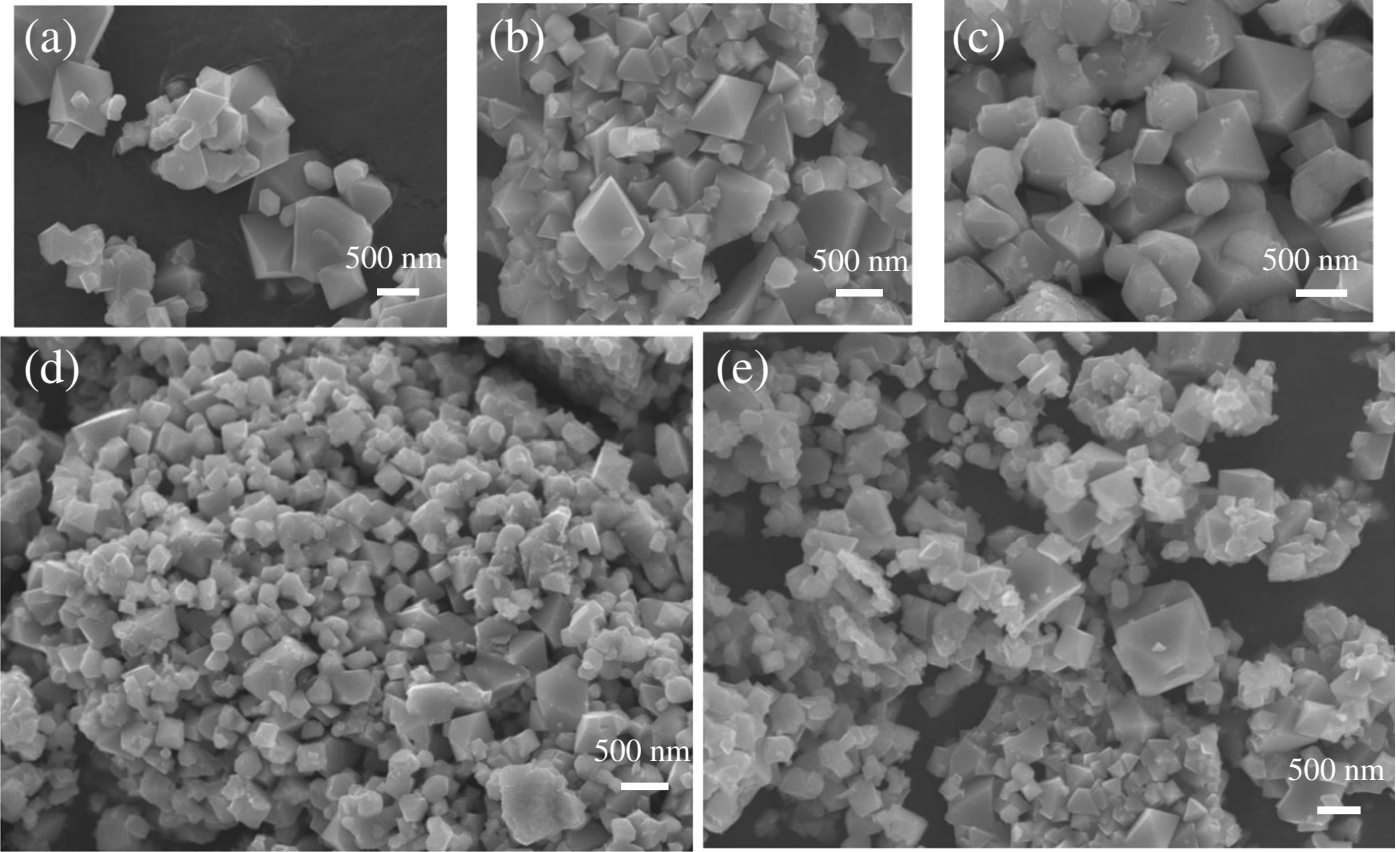
SEM images of the LiNi_0.5-x_Ga_x_Mn_1.5_O_4_ (*x* = 0, 0.04, 0.06, 0.08, 0.1) **a** Ga-0, **b** Ga-0.04, **c** Ga-0.06, **d** Ga-0.08, and **e** Ga-0.10

**Fig. 4:**
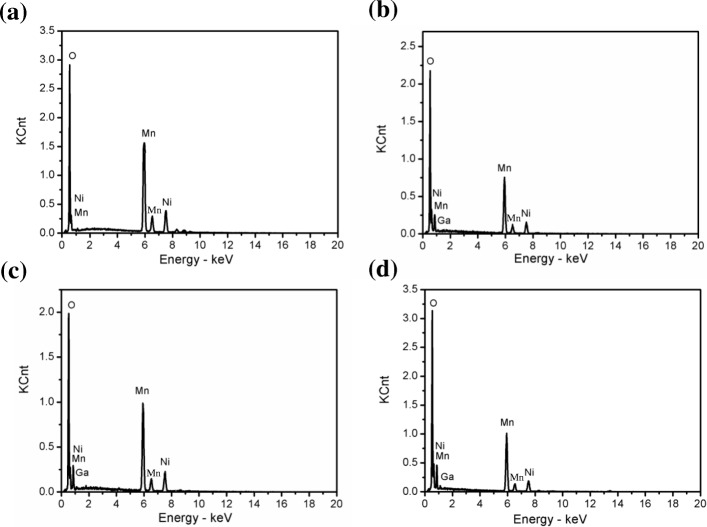
EDS image of the LiNi_0.5-x_Ga_x_Mn_1.5_O_4_ (*x* = 0, 0.04, 0.06, 0.08, 0.1) **a** Ga-0, **b** Ga-0.04, **c** Ga-0.06, and **d** Ga-0.08

The particle morphologies of the samples are observed by SEM. The results, as shown in Fig. 3, imply that all samples have a spinel octahedron structure and possess a fine crystal. Some particles could be observed on the surface of the Ga-doped samples, but were absent in LiNi_0.5_Mn_1.5_O_4_. As shown in Fig. 4, EDS is a method of qualitative analysis which illustrates the presence of Ga in Ga-doped samples. Obviously, following the addition of *x* value, a significant increase in the concentration of Ga was recorded, indicating that Ga had been doped into the crystal lattice.

### Electrochemical performance analysis

**Fig. 5:**
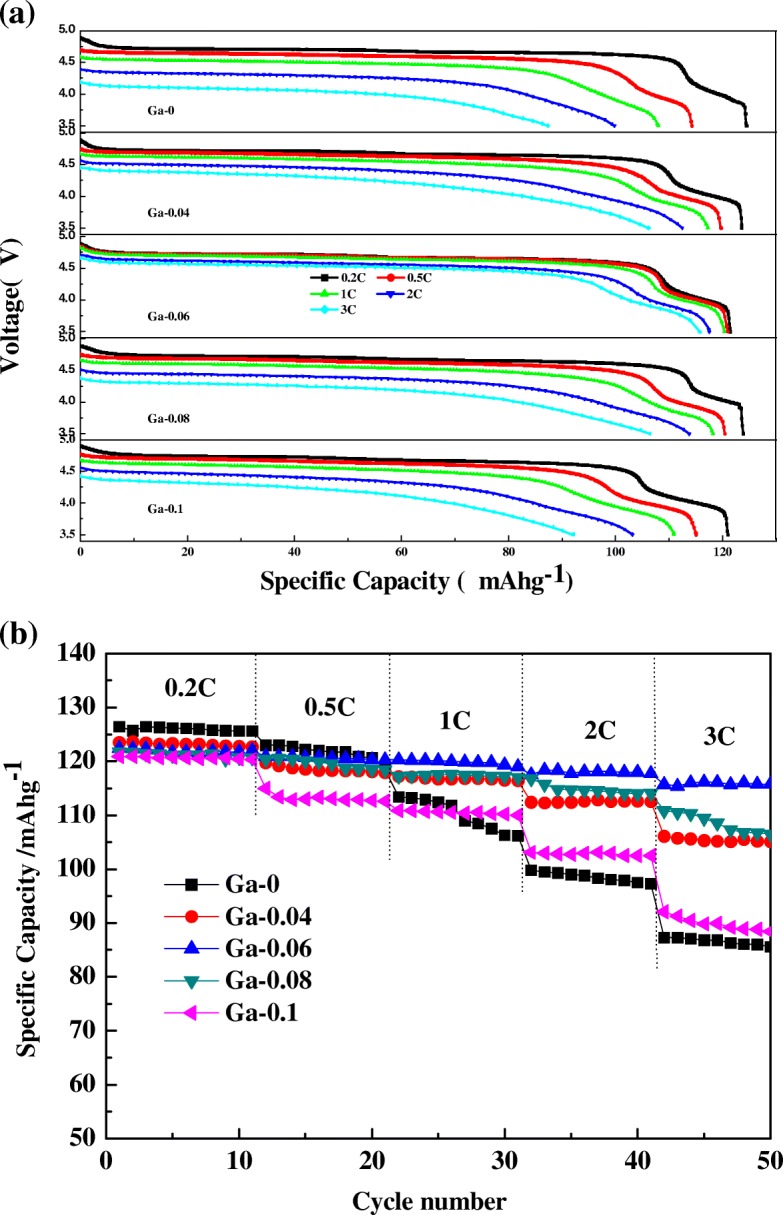
**a** The discharge curves of LiNi_0.5-x_Ga_x_Mn_1.5_O_4_ (*x* = 0, 0.04, 0.06, 0.08, 0.1) samples at 0.2 C, 0.5 C, 1 C, 2 C, 3 C rates. **b** Rate capabilities of the LiNi_0.5-x_Ga_x_Mn_1.5_O_4_ (*x* = 0, 0.04, 0.06, 0.08, 0.1) samples

To examine the impacts of Ga doping on improving the rate capability of LiNi_0.5-x_Ga_x_Mn_1.5_O_4_ (*x* = 0, 0.04, 0.06, 0.08, 0.1), the capacities of the pristine and Ga-doped samples at 0.2, 0.5, 1, 2, and 3 C discharge rates were investigated. From Fig. 5a, rate capability was obviously promoted after Ga doping. It is notable that Ga-0.06 achieved an outstanding rate performance, 122.5, 120.9, 120.3, 117.5, 115.7 mAh/g at rates of 0.2, 0.5, 1, 2, and 3 C, respectively, compared to the 124.4, 114.2, 108, 99.8, 87.3 mAh/g of LiNi_0.5_Mn_1.5_O_4_ at the same rates. The discharge capacity of doped samples was lower than the pristine one at a 0.2 C discharge rate as a consequence of the electrochemically active Ni^2+^ that has been substituted with the Ga. For the discharge plateaus, the most obvious finding to emerge from Fig. 5a is that two discharge plateaus at ~ 4.0 V and ~ 4.7 V could be observed in accordance with Mn^3+^/Mn^4+^ and Ni^2+^/Ni^4+^ redox couples, which means that Ga doping does not modify the discharge mechanism. Figure 5b shows the rate capability curves of the LiNi_0.5-x_Ga_x_Mn_1.5_O_4_ (*x* = 0, 0.04, 0.06, 0.08, 0.1) samples. However, the discharge capacity of the pristine sample decreases rapidly with increasing C-rates. The excellent rate capability of Ga-0.06 can be ascribed to the reduced Li_x_Ni_1-x_O impurity phase, improved electronic conductivity, and the improved diffusion coefficient of Li^+^. The impurity phase would hinder the Li^+^ ions from taking off or embedding. The electrical conductivity was improved as a result of the increase of the Mn^3+^ content by Ga doping. This finding is in agreement with the dQ/dV plots. There are two sources of Mn^3+^; one source of Mn^3+^ is oxygen deficiency [24], resulting in Mn^3+^, while another is the substitution of Ga^3+^ for Ni^2+^ in which quite a few portions of Mn^4+^ should transform into Mn^3+^ to maintain charge neutrality. However, the disproportionation reaction of Mn^3+^ that occurs in the electrolyte is not conducive to structural stability. Simultaneously, the doped Ga formed a passivation layer and reduced the direct contact between the electrolyte and the electrode material. This inhibited the occurrence of disproportionation, leading to excellent rate properties. All of the above analysis is also in accordance with the SEM and EDS results.

**Fig. 6:**
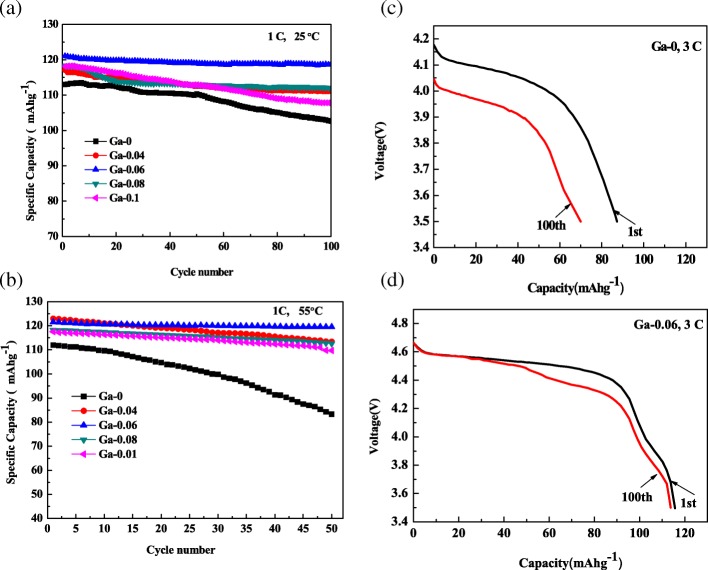
**a** Cycle performance of the LiNi_0.5-x_Ga_x_Mn_1.5_O_4_ (*x* = 0, 0.04, 0.06, 0.08, 0.1) samples at 1 C and 25 °C, **b** cycle performance of the LiNi_0.5-x_Ga_x_Mn_1.5_O_4_ (*x* = 0, 0.06) samples at 1 C and 55 °C, **c** discharge curves of the Ga-0 samples, and **d** Ga-0.06 sample at 3 C

The cycle performance of the cell is an essential parameter for electrochemical properties. From Fig. 6a, we calculated that the capacity retention at 1 C and 25 °C of the Ga-0, Ga-0.04, Ga-0.06, Ga-0.08, and Ga-0.1 samples are 90.8, 94.9, 98, 94.6, and 91.2%, respectively. The cycling performance clearly improved to different degrees after Ga doping, and Ga-0.06 samples showed the highest performance parameters. Figure 6b shows the cycle performance of the Ga-0, Ga-0.04, Ga-0.06, Ga-0.08, and Ga-0.1 samples at 1 C and 55 °C. The capacity retention of the Ga-0.06 samples was 98.4% of its initial capacity (121.5 mAh/g) at 1 C and 55 °C after 50 cycles, but the Ga-0 sample delivered a discharge capacity of 113 mAhg^−1^ and faded sharply, with a capacity retention of 74% at the 50th cycle. Consequently, the Ga-0.06 samples are better than that Ga-0 samples for improving the cycle stability at a high temperature, which should be ascribed to the reduced Li_x_Ni_1-x_O impurity phase and the stable structure provided by the passivation effect resulting from Ga doping. Figure 6c, d provides the discharge curves of the Ga-0 and Ga-0.06 composites at 3 C. The capacity retention of the Ga-0.06 sample reached 98.3% after 100 cycles at 3 C, which was higher than that of the pristine sample (80%). The discharge plateau at 3 C of the pristine sample was lower than that of Ga-0.06, which implied that the degree of polarization of the pristine sample was larger than that of Ga-0.06. It can be concluded that appropriate Ga-doping content is beneficial to the enhancement of the electrochemical properties, especially for the cycle stability at high temperatures and high discharge rates.

**Fig. 7:**
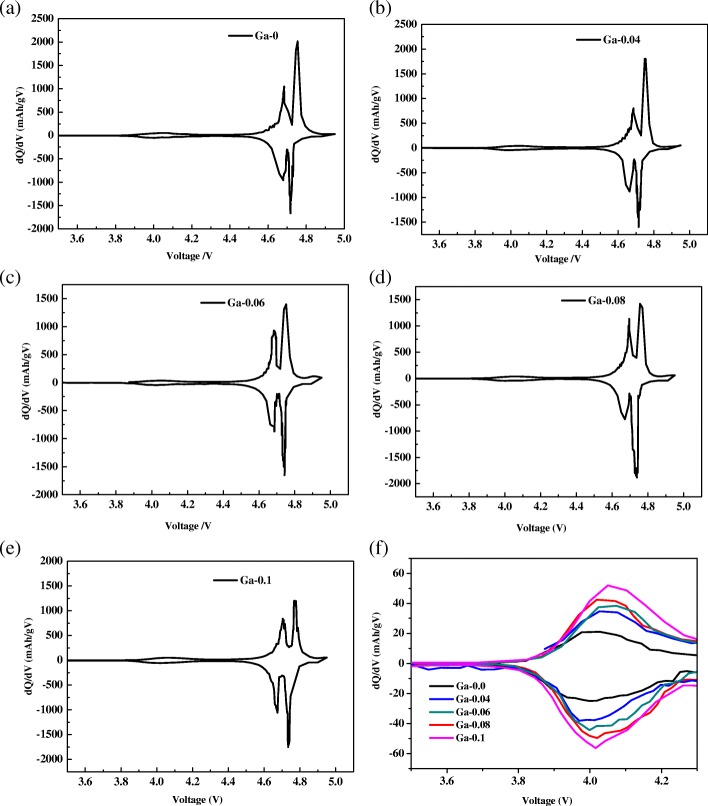
**a**~**e** dQ/dV plots of the LiNi_0.5-x_Ga_x_Mn_1.5_O_4_ (*x* = 0, 0.04, 0.06, 0.08, 0.1) samples; **f** the enlarged dQ/dV plots between 3.5 and 4.3 V

For more detailed analysis of the electrochemical behavior, the dQ/dV plots are represented in Fig. 7a–e. The peak at approximately 4.0 V is shown in Fig. 7f, which should be assigned to Mn^3+^/Mn^4+^ redox couple [25], indicating the characteristics of the disordered Fd3m spinel structure [9]. The two separating peaks are at approximately 4.7 V, corresponding to Ni^2+^/Ni^3+^ and Ni^3+^/ Ni^4+^ redox couples [26]. It is clear that the intensity of the peak at approximately 4.7 V tended to decrease with the content of Ga, which is caused by the substitution of electrically active Ni by Ga. The intensity of the peak at approximately 4.0 V increased, which is attributed to the concentration of Mn^3+^ ions increasing with the content of Ga. The smaller the potential difference between the redox peak and oxidation peak, the weaker the polarization. The degree of polarization is an indicator of the reversibility of Li^+^ ions in the electrode. From Fig. 7a–e, we determined that the smallest voltage difference between oxidation and reduction peaks of Ni^3+^/Ni^4+^ redox couples is 0.011 V for the Ga-0.06 sample, which is lower than that of the pristine sample (0.037 V), reflecting the best reversibility of Li^+^ ion insertion and de-insertion in the electrode. The analysis results of dQ/dV plots indicated that an appropriate Ga doping content has a positive effect on the reversibility of the samples. This finding is in good accordance with the results of rate capacity and *D*_Li_^+^ shown in Table 3.

**Table 3:** Lithium ion diffusion coefficient (*D*_Li_^+^) for LiNi_0.5-x_Ga_x_Mn_1.5_O_4_ (*x* = 0, 0.04, 0.06, 0.08, 0.1) samples obtained from EIS

Samples (LiNi_0.5-x_Ga_x_Mn_1.5_O_4_)	*R*_ct_ (Ω)	σ (Ωs^−1/2^)	*D*_Li_^+^ (cm^2^ s^−1^)
Ga-0	168.40	155.01	3.89 × 10^−12^
Ga-0.04	133.00	115.64	6.99 × 10^−12^
Ga-0.06	86.73	34.22	7.99 × 10^−11^
Ga-0.08	113.30	43.77	4.88 × 10^−11^
Ga-0.1	143.66	105.32	8.43 × 10^−12^

**Fig. 8:**
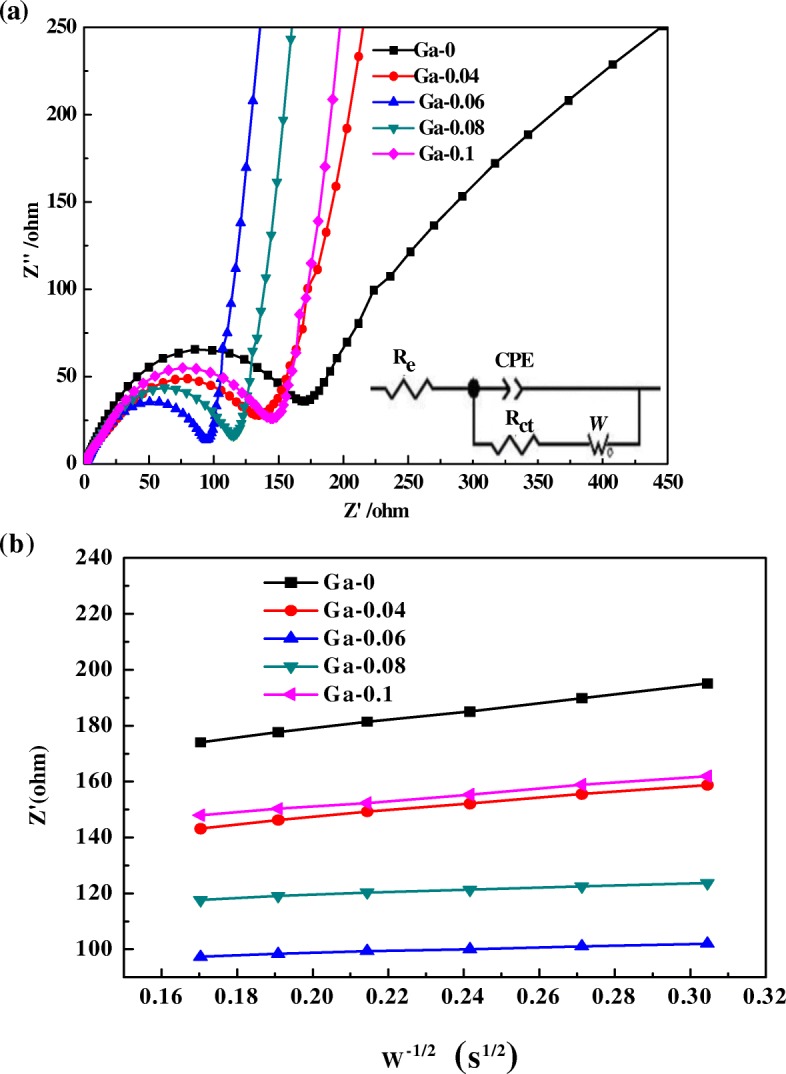
**a** EIS spectra of the LiNi_0.5-x_Ga_x_Mn_1.5_O_4_ (*x* = 0, 0.04, 0.06, 0.08, 0.1) samples. **b** Graph of Z’ plotted against ω^-1/2^ in the low-frequency region for the LiNi_0.5-x_Ga_x_Mn_1.5_O_4_ (*x* = 0, 0.04, 0.06, 0.08, 0.1) samples

To investigate the impact of Ga doping on the electrochemical reaction kinetics more deeply, Fig. 8a provides the EIS spectra of the obtained samples after 3 cycles at a rate of 0.1 C. The Nyquist plots and equivalent circuits (inset) of the LiNi_0.5-x_Ga_x_Mn_1.5_O_4_ (*x* = 0, 0.04, 0.06, 0.08, 0.1) composites are presented in Fig. 8a. CPE corresponds to the constant phase element of the double-layer, *R*_e_ indicates the solution resistance, and *R*_ct_ stands for charge transfer impedance, which is described by the diameter of a semicircle. *W* stands for the Warburg impedance, which reflects a speed of lithium-ion diffusion. We can determine that the *R*_ct_ of the LiNi_0.5-x_Ga_x_Mn_1.5_O_4_ (*x* = 0, 0.04, 0.06, 0.08, 0.1) samples are 168.4, 133, 86.73, 113.3, 143.66 Ω, respectively (as shown in Table 3). The *R*_ct_ decreased along with the concentration of Ga doping, and the minimum *R*_ct_ value occurred for the Ga doping content of 0.06, indicating an enhancement of electrochemical reaction kinetics. The lower *R*_ct_ value of the Ga-0.06 samples reflects the lower electrochemical polarization, which is in line with the dQ/dV plots. The diffusion coefficient of Li^+^ (*D*_Li_^+^) is obtained from the following equation [27]:

1 $$ {D}_{L{\mathrm{i}}^{+}}=\frac{R^2{T}^2}{2{A}^2{n}^4{F}^4{C}_{L{i}^{+}}^2{\sigma}^2} $$

In this equation, *R* represents the gas constant, (*R* = 8.314 JK mol^−1^), *T* stands for the temperature (298 K), *A* corresponds to the surface area of the electrode, *n* stands for the number of electrons per molecule attending the electronic transfer reaction, *F* stands for the Faraday constant (*F* = 96,500 C mol^−1^), *C*_Li_^+^ is the lithium ions content in samples, and σ is the Warburg factor. A relationship between σ and Z’ is listed in Eq. (2) and was determined from the slope of the line of the low frequency zone in Fig. 8b, (as listed in Table 3).

2 $$ {Z}^{\hbox{'}}={R}_{\mathrm{e}}+{R}_{ct}+\sigma {\omega}^{-1/2} $$

It is clear that there has been an increase and then decrease in the *D*_Li_^+^, which was the opposite of charge transfer impedance (*R*_ct_). The *D*_Li_^+^ values are 3.89 × 10^−12^, 6.99 × 10^−12^, 7.99 × 10^−11^, 4.88 × 10^−11^, 8.43 × 10^−11^ cm^2^ s^−1^ for Ga-0, Ga-0.04, Ga-0.06, Ga-0.08, Ga-0.1, respectively. The difference in the *D*_Li_^+^ between Ga-doped and pristine samples amount to 1 order of magnitude, indicating that Ga doping is a good way to enhance the ionic conductivity. The lowest charge transfer impedance and the highest diffusion coefficient of Li^+^ of Ga-0.06 gave it excellent cycling and rate properties compared to all the samples. The increase of *D*_Li_^+^ can be ascribed to the reduced Li_x_Ni_1-x_O impurity phase. These results indicate that an appropriate Ga-doping content can not only improve the conductivity of the LNMO but also enhance the diffusion coefficient of Li^+^.

## Conclusions

A sol-gel method was utilized to synthesize LiNi_0.5-x_Ga_x_Mn_1.5_O_4_ (*x* = 0, 0.04, 0.06, 0.08, 0.1) samples. All samples have a disordered Fd3m structure and possess a fine spinel octahedron crystal. Ga doping restrained the formation of the Li_x_Ni_1-x_O secondary phase and increased the degree of cation disorder. The outstanding performance should be attribute to the enhanced conductivity, reduced electrochemical polarization and the passivation layer by the Ga doping, which is more pronounced at high rates and high temperatures. Furthermore, the Ga-0.06 sample with an optimum content of Ga exhibits excellent electrochemical performance compared to the other samples; the capacity retention at 1 C and 55 °C of the Ga-0.06 sample was 98.4% of its initial capacity (121.5 mAh/g) after 50 cycles, but the Ga-0 sample delivered a discharge capacity of 113 mAhg^−1^ and faded sharply, with a capacity retention of 74% at the 50th cycle under the same test conditions. Our work provides a promising concept for improving the cycle stability of the cathode materials of Li-ion batteries at high temperatures.

## Methods

### Material syntheses

LiNi_0.5-x_Ga_x_Mn_1.5_O_4_ (*x* = 0, 0.04, 0.06, 0.08, 0.1) was synthesized by a sol-gel method. The raw materials are listed as follows: CH_3_COOLi·2H_2_O (99.9%, Aladdin), Mn(CH_3_COO)_2_·4H_2_O (98%, Tianjin Damao), Ni(CH_3_COO)_2_·4H_2_O (99.9%, Aladdin), Ga(NO_3_)_3_·xH_2_O (99.9%, Aladdin), citric acid (99.5%, Aladdin), and ammonium hydroxide (25%, Tianjin Damao). The synthetic steps are shown below. Firstly, a certain stoichiometric ratio of CH_3_COOLi·2H_2_O, Mn(CH_3_COO)_2_·4H_2_O, Ni(CH_3_COO)_2_·4H_2_O, and Ga(NO_3_)_3_·xH_2_O was dissolved in a certain quality of distilled water under vigorous agitation at room temperature. Over 5% CH_3_COOLi·2H_2_O was added to make up for the loss of lithium salt. Secondly, a certain amount of citric acid was added to the above solution in a water bath with stirring at 80 °C. Thirdly, ammonium hydroxide was used to adjust the pH of the mixture to 7, and agitation was continued until a gel was obtained. Finally, the resulting gel was dried at 110 °C in a vacuum oven for 10 h. The dried precursors were pre-calcined at 650 °C for 5 h, grinding it into powder, and further calcined at 850 °C for 16 h in a muffle furnace. Samples with different Ga doping contents were obtained after cooling to room temperature, for the sake of convenience denoted as Ga-0, Ga-0.04, Ga-0.06, Ga-0.08, Ga-0.1, respectively.

### Materials characterization

X-ray diffraction (XRD, Cu Kα, 36 kV, 20 mA) was employed on a Rigaku D/max-PC2200 system to assess the structure of the samples over a range from 10 to 80° at 4°/min. The Fourier transform infrared spectra (FT-IR) were measured by a Nicoletis 6700 instrument. Scanning electron microscopy (SEM, JEOL JMS-6700F) was used to record the morphology of the composites. Elemental composition was analyzed using energy dispersive spectrometry (EDS) along with SEM.

### Electrochemical measurements

The electrochemical performance of the samples was evaluated by CR2032 coin cells. To prepare working electrodes, 90 wt% LiNi_0.5-x_Ga_x_Mn_1.5_O_4_ (*x* = 0, 0.04, 0.06, 0.08, 0.1) samples, 5 wt% super P conductive agent, and 5 wt% polypropylene fluoride (PVDF) binder were dissolved into *N*-methyl-2-pyrrolidone (NMP) to form a homogeneous slurry. The obtained slurry was cast onto an aluminum foil and dried under vacuum at 85 °C overnight. Then, the foil was pressed and cut into disks with a diameter of 14 mm. CR2032 coin cells with lithium foil as the counter and reference electrodes were used to assess the electrochemical performance of the materials, and it was assembled in an argon-filled glove box in which both the content of water and oxygen levels were kept below 0.1 ppm. Here, the electrolyte with high voltage resistance was 1 M LiPF_6_ in an ethylene carbonate (EC), propylene carbonate (PC), and ethylene methyl carbonate (EMC) mixture (EC:PC:EMC = 1:2:7, *v*:*v*:*v*). Galvanostatic charge-discharge measurements were carried out at 25 °C and 55 °C at a voltage of 3.5–4.95 V by the LAND battery testing system. Electrochemical impedance spectroscopy (EIS) tests were performed on a CHI600A electrochemical workstation. EIS spectroscopy in the frequency range of 0.01 Hz to 100 kHz with a perturbation of 5 mV was carried out.

## Abbreviations

A: Surface area of the electrode
C_Li_^+^: Lithium ions content
CPE: Constant phase
CV: Cyclic voltammetry
*D*_Li_^+^: Diffusion coefficient of Li^+^
EC/PC/EMC: Ethylene carbonate/propylene carbonate/ethylene methyl carbonate
EDS: Energy dispersive spectrometry
EIS: Electrochemical impedence spectroscopy
F: Faraday constant
FT-IR: Fourier transform spectrophotometer
Ga-0.04: LiNi_0.46_Ga_0.04_Mn_1.5_O_4_
Ga-0.06: LiNi_0.44_Ga_0.06_Mn_1.5_O_4_
Ga-0.08: LiNi_0.42_Ga_0.08_Mn_1.5_O_4_
Ga-0.1: LiNi_0.4_Ga_0.1_Mn_1.5_O_4_
I_311_: The intensity of (311) diffraction peak
I_400_: The intensity of (400) diffraction peak
I_588_: The intensity of 588 cm^−1^ band
I_621_: The intensity of 621 cm^−1^ band
LNMO/Ga-0: LiNi_0.5_Mn_1.5_O_4_
*n*: The number of electrons per molecule
NMP: *N*-methyl-2-pyrrolidinone
PVDF: Polyvinylidene fluoride
R: Gas constant
R_ct_: Charge transfer resistance
R_e_: Solution resistance
SEM: Scanning electron microscope
T: Temperature
*W*: Warburg impedance
XRD: X-ray diffraction
σ: The Warburg factor
